# Supplementary figures and images for: A nuclear function for an oncogenic microRNA as a modulator of snRNA and splicing
Source: Mol Cancer. 2022 Jan 15;21:17. doi: 10.1186/s12943-022-01494-z (PMC8760648; doi:10.1186/s12943-022-01494-z)

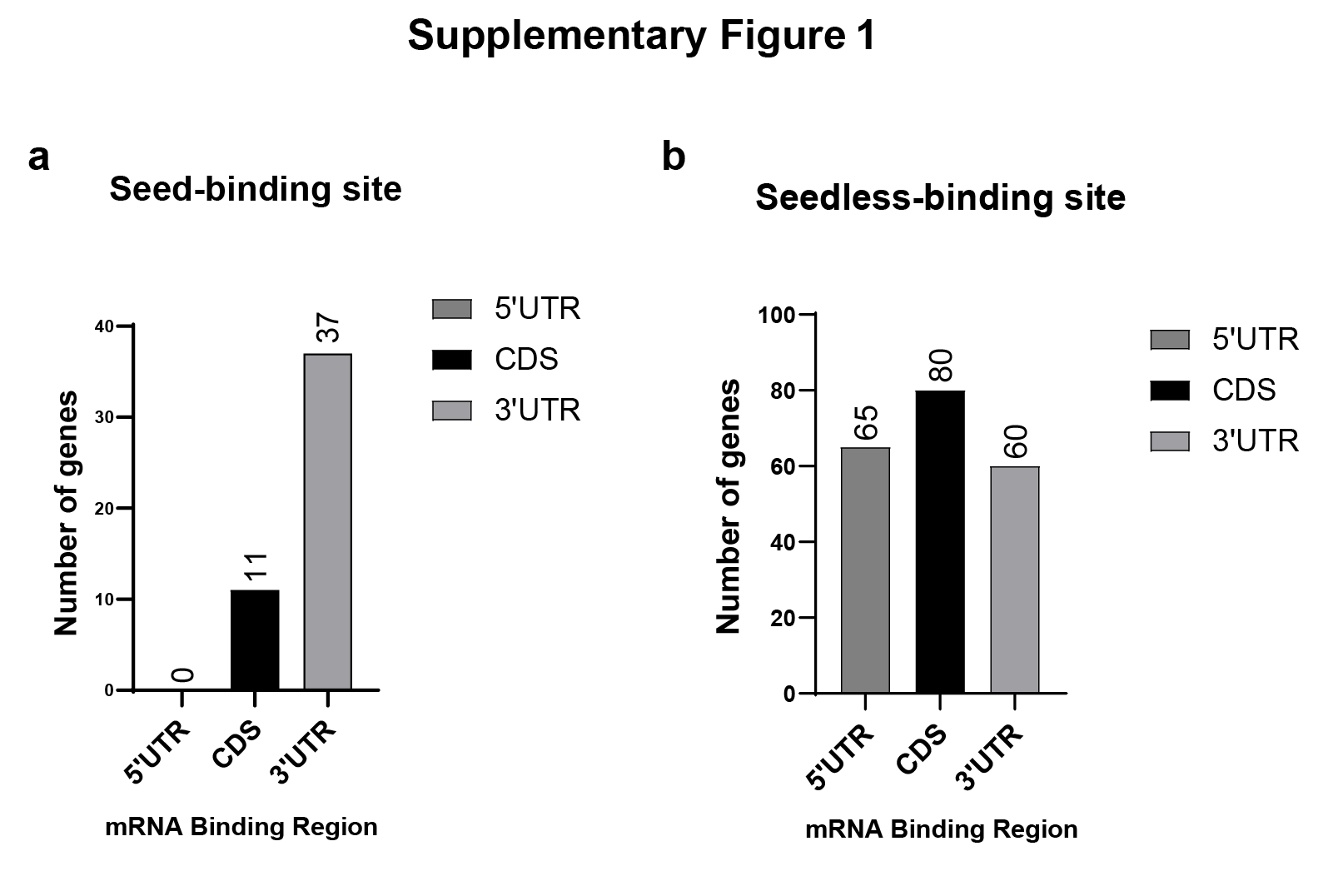

Supplement: Supplementary file 1 — Additional file 1 Supplementary Fig. 1. Analysis of putative miR-10b binding sites in 5′ UTR, CDS, and 3′ UTR of mRNA targets identified by CLEAR-CLIP, using a STarMirDB tool [66], demonstrates the distribution of seed-based and seedless targets. Related to Fig. 1. [file 12943_2022_1494_MOESM1_ESM.tif]

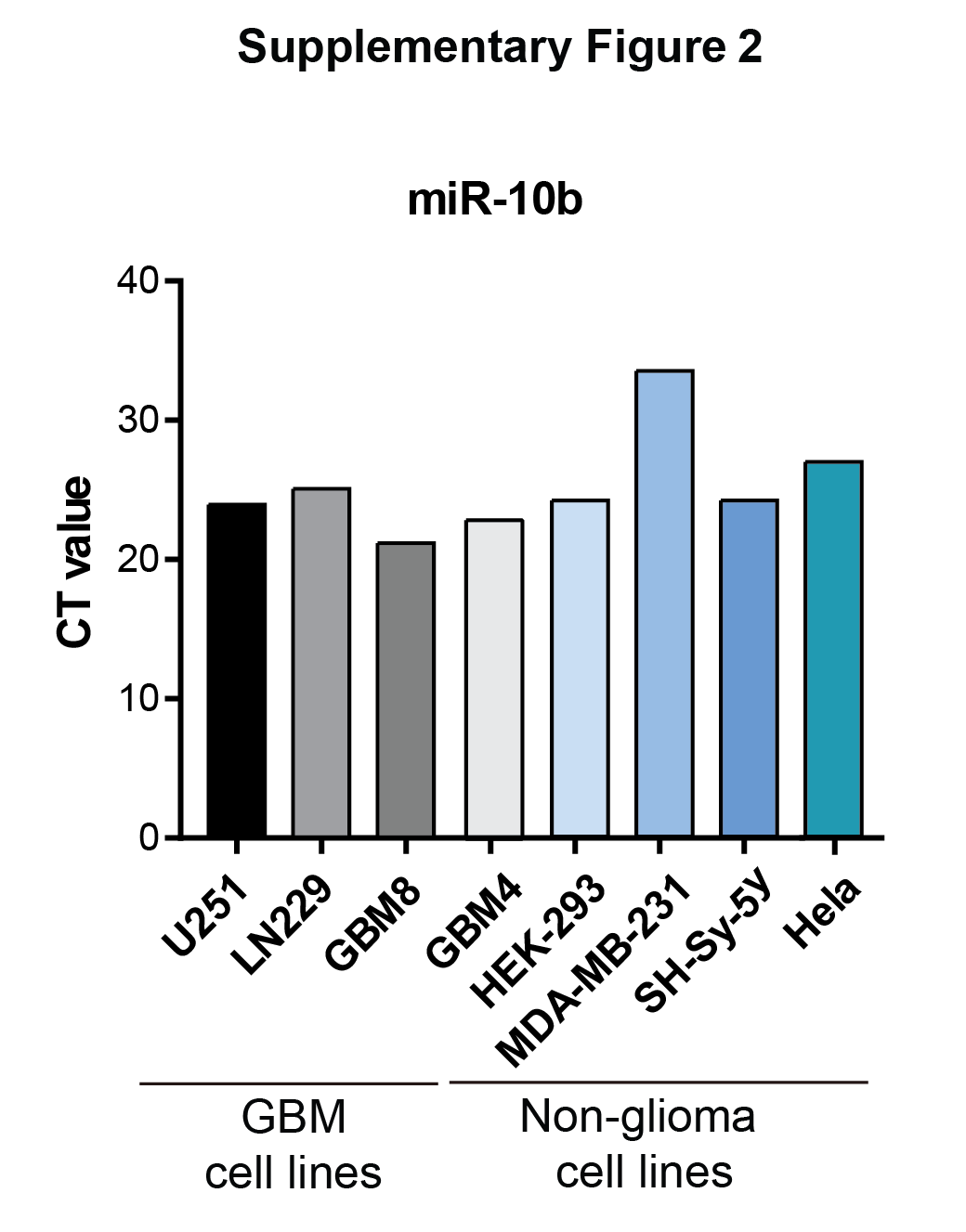

Supplement: Supplementary file 2 — Additional file 2 Supplementary Fig. 2. Relative levels of miR-10b in glioma and non-glioma cell lines. The qRT-PCR reactions were performed with equal RNA input and the Ct values are indicated. [file 12943_2022_1494_MOESM2_ESM.tif]

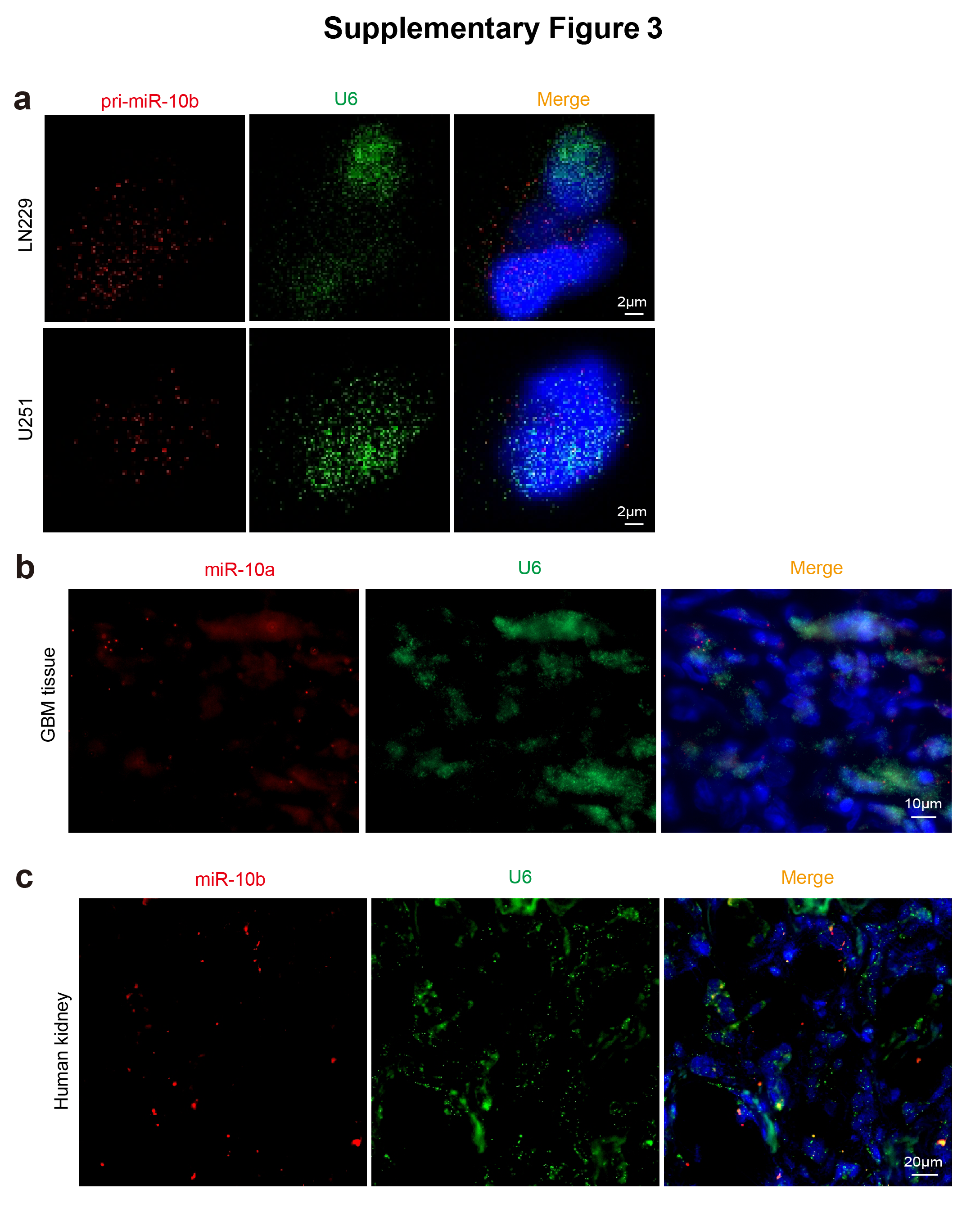

Supplement: Supplementary file 3 — Additional file 3 Supplementary Fig. 3. Additional data for miR-10b and U6 FISH in cells and tissues. a Representative FISH images of pri-miR-10b (red) and U6 (green) in cultured LN229 and U251 glioma cells with the corresponding fluorescently labeled probes, and nuclei stained with DAPI. b Representative FISH images of the miR-10b paralogue miR-10a (red) and U6 (green) in patient-derived GBM tissues demonstrate low miR-10a expression and lack of colocalization. c Representative FISH images of miR-10b (red) and U6 (green) in human kidney tissues. [file 12943_2022_1494_MOESM3_ESM.tif]

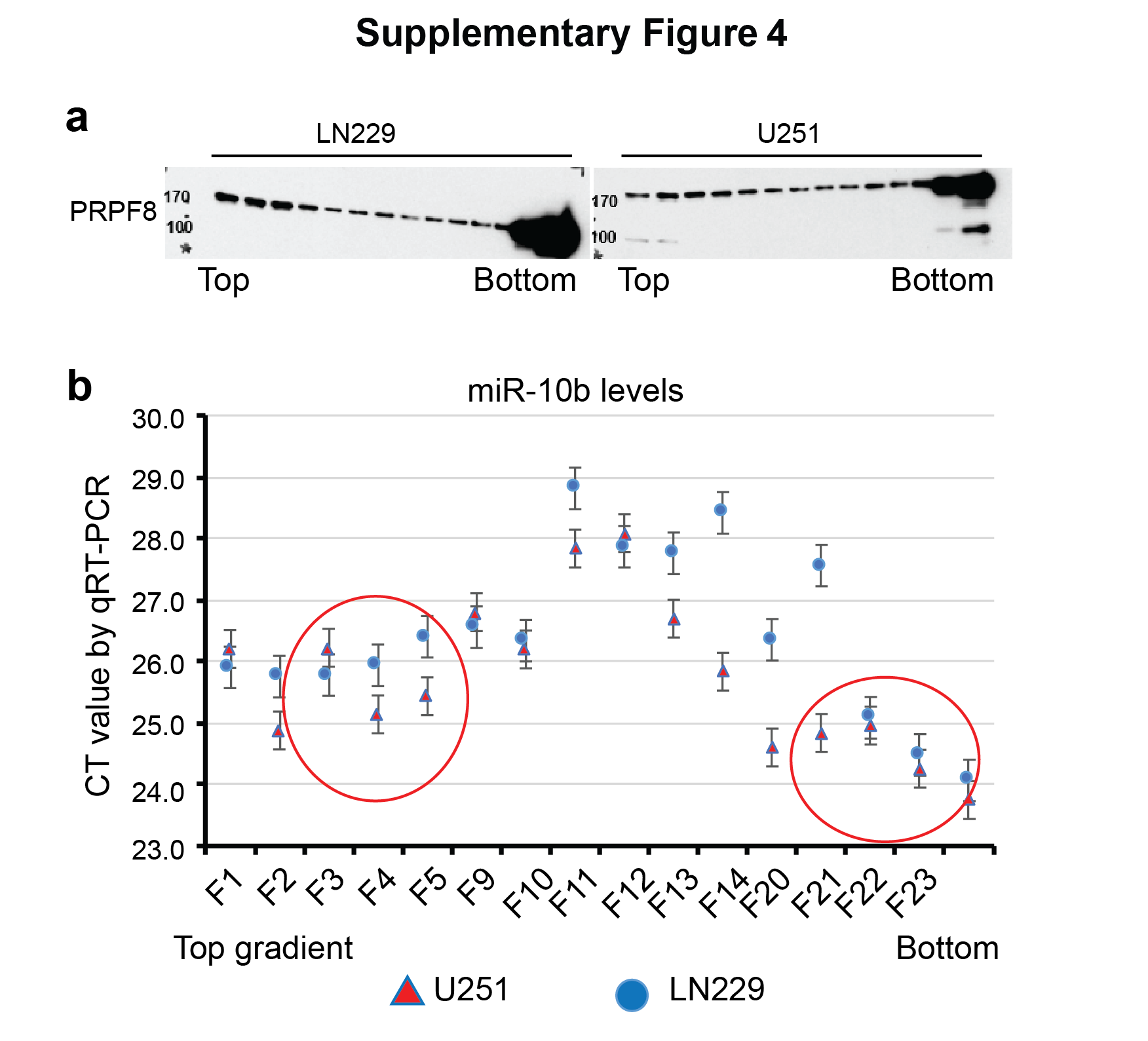

Supplement: Supplementary file 4 — Additional file 4 Supplementary Fig. 4. MiR-10b is enriched in the spiceosome in glioma cells. a Western blot analysis of LN229 and U251 nuclear fractions separated in glycerol gradient, demonstrates enrichment of the spliceosomal marker PRPF8 in the heavy fractions. b qRT-PCR analysis demonstrates miR-10b enrichment (Ct values < 25.5) in the corresponding heavy fractions in both cell lines. [file 12943_2022_1494_MOESM4_ESM.tif]

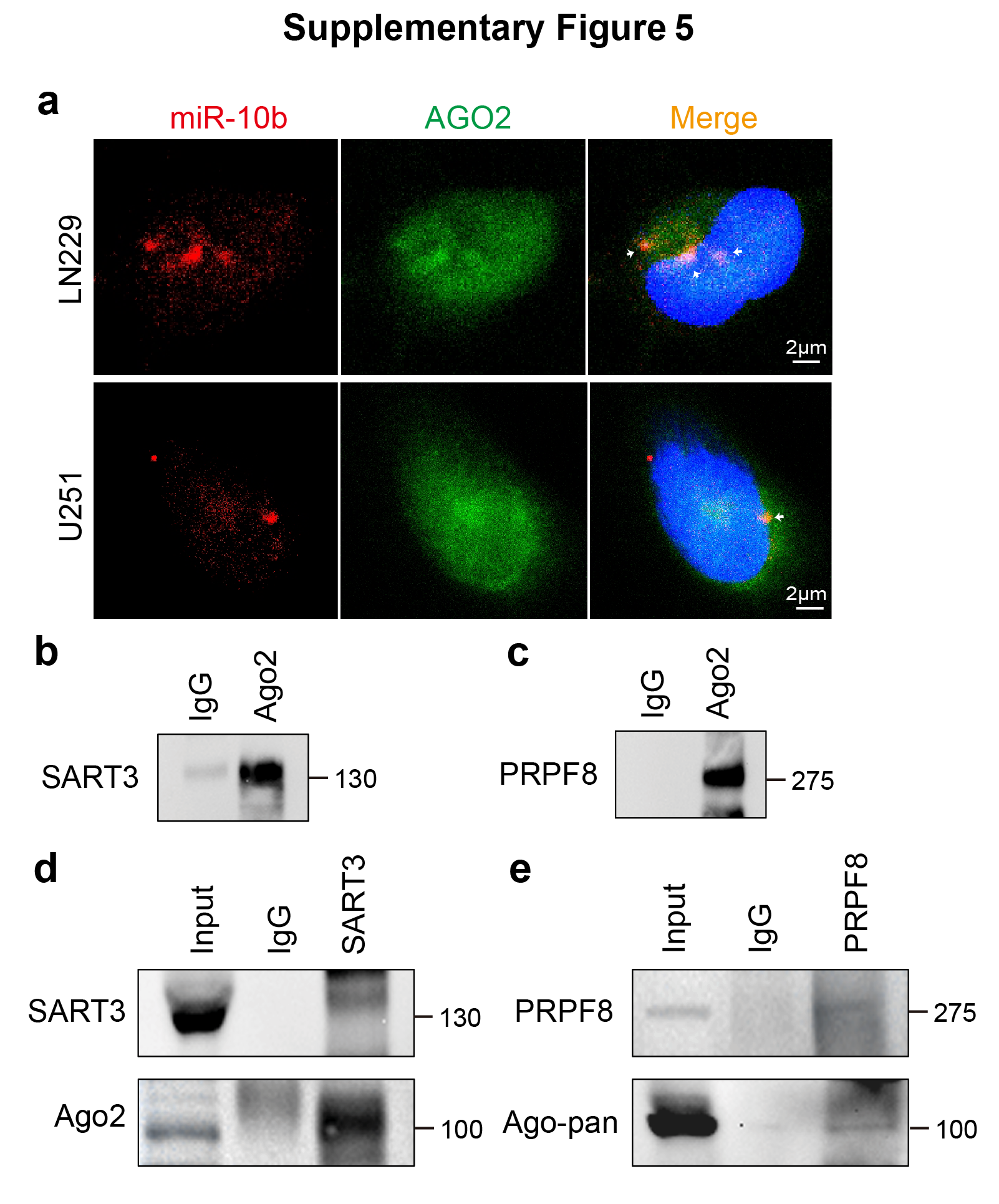

Supplement: Supplementary file 5 — Additional file 5 Supplementary Fig. 5. SART3 and PRPF8 bind to AGO proteins in glioma cells. a Representative images of miR-10b FISH (red) and AGO2 immunofluorescence (green) in glioma cells, and nuclei stained with DAPI (blue). Arrows mark the colocalization of AGO2 and miR-10b. b-e SART3 and PRPF8 bind to AGO proteins in glioma cells. IP with either IgG (control), AGO2, SART3, or PRPF8 antibodies, followed by the Western blotting for the indicated proteins. [file 12943_2022_1494_MOESM5_ESM.tif]

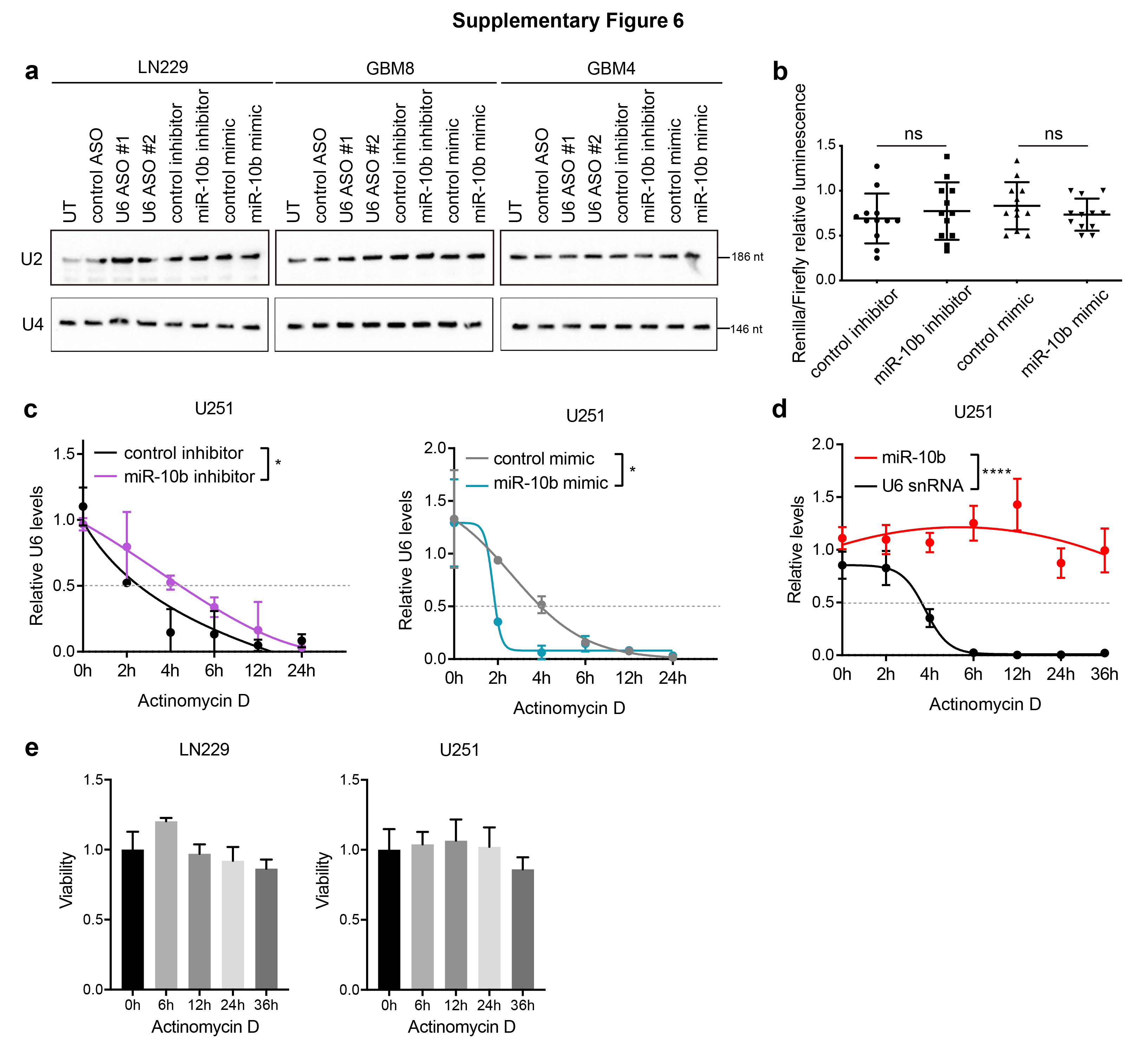

Supplement: Supplementary file 6 — Additional file 6 Supplementary Fig. 6. miR-10b regulates U6 snRNA but not U2 and U4 snRNAs in glioma cells. a Representative denaturing Northern blotting of glioma cells and GSCs transfected with either U6 ASOs, miR-10b inhibitor or mimic, with probes specific for U2 and U4 snRNAs. b miR-10b mimic and inhibitor do not regulate 3′ UTR luciferase reporters bearing full-length U6 snRNA sequence in glioma cells. The data is presented as Renilla/Firefly relative luminescence and normalized to the corresponding values in cells not transfected with the oligonucleotides. n = 12; Graphical data are shown as mean ± SEM. c qRT-PCR analysis of snRNA U6 levels in U251 cells transfected with either miR-10b inhibitor, mimic, or corresponding controls, and treated with 5 μg/ml Actinomycin D (mean ± SD, n = 3). P values were calculated using two-way ANOVA. d U251 cells were treated with Actinomycin D followed by the qRT-PCR analysis of miR-10b and U6 levels (mean ± SD, n = 3). P values were calculated using two-way ANOVA. e LN229 and U251 cells were treated with Actinomycin D followed by WST-1 analysis at the indicated time points (mean ± SD, n = 3). P values were calculated using two-tail unpaired t-test. * P < 0.05; **** P < 0.0001. [file 12943_2022_1494_MOESM6_ESM.tif]

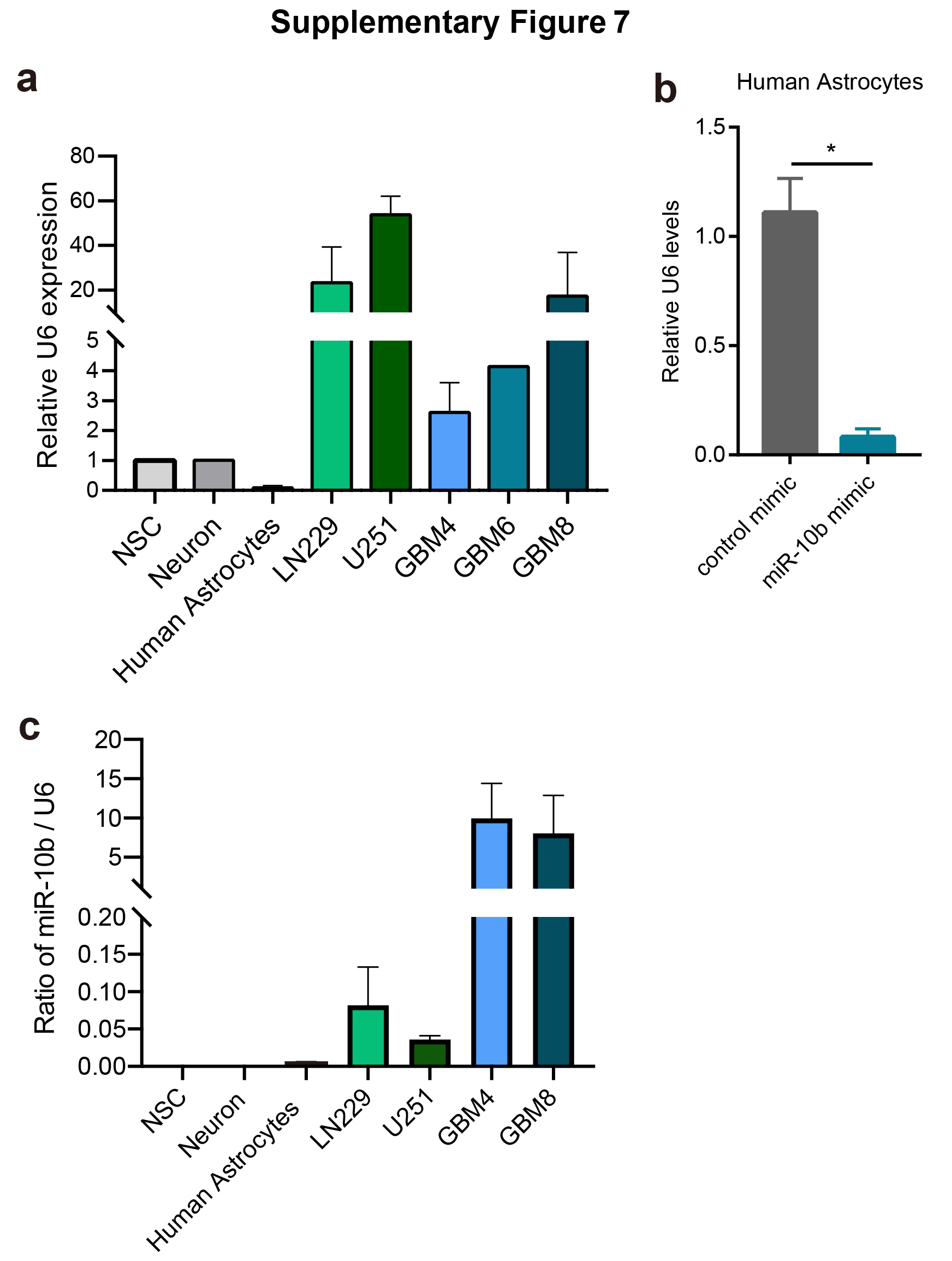

Supplement: Supplementary file 7 — Additional file 7 Supplementary Fig. 7. Relative levels of U6 snRNA in normal neuroglial and glioma cells. a qRT-PCR analysis of U6 levels in normal human neuroglial cells (neuroprogenitors (NSC), neurons, and astrocytes) and glioma cells. The expression of U6 was normalized by uniformly expressed miR-125a. b qRT-PCR analysis of U6 levels in human astrocytes transfected with miR-10b mimic, or corresponding control oligonucletides (mean ± SD, n = 3). P values were calculated using two-tail unpaired t-test. c miR-10b and U6 levels in normal human neuroglial cells and glioma cells were detected by qRT-PCR. The expression of miR-10b and U6 was normalized by miR-125a and relative miR-10b/U6 ratios were presented. * P < 0.05. [file 12943_2022_1494_MOESM7_ESM.tif]

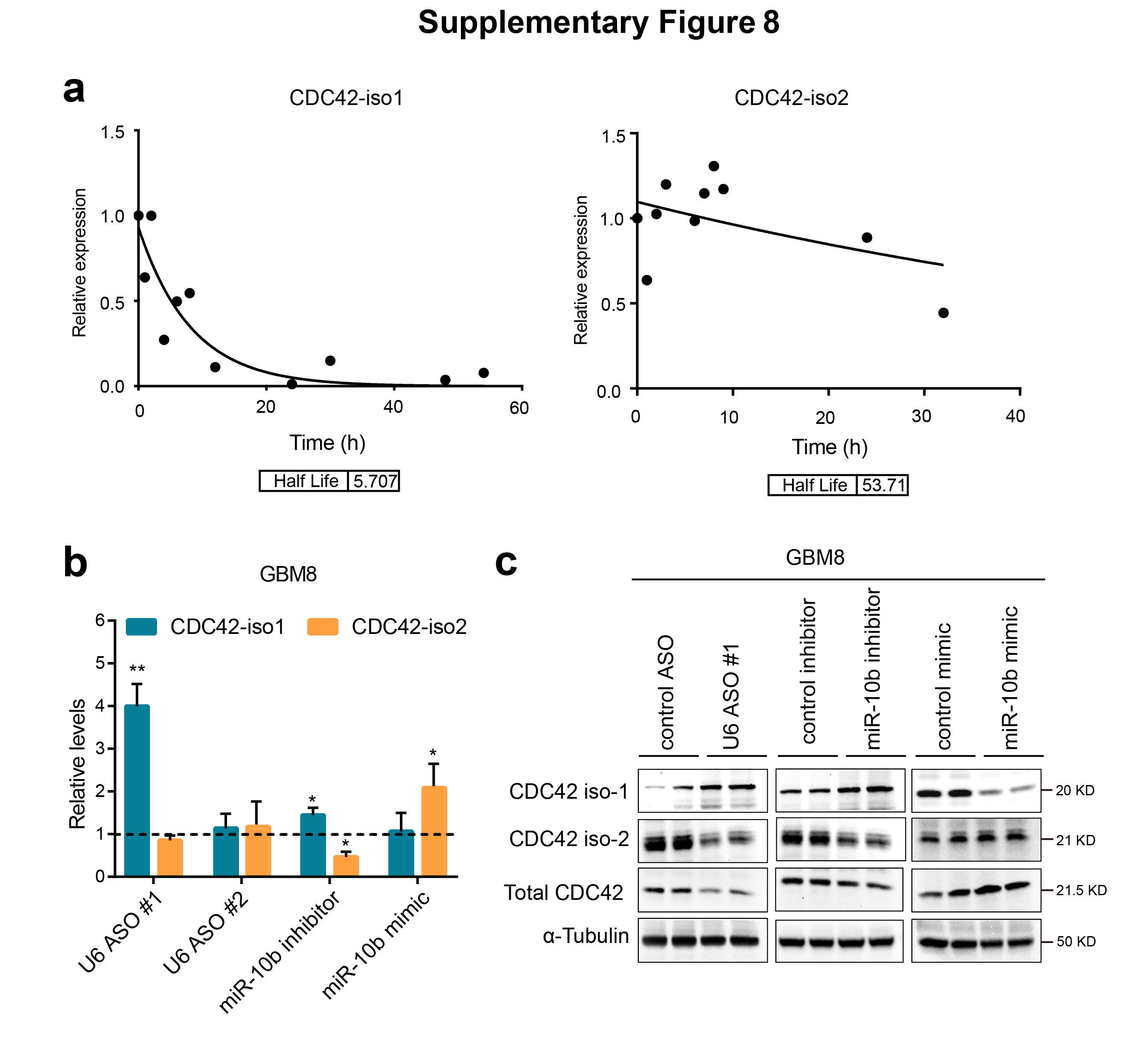

Supplement: Supplementary file 8 — Additional file 8 Supplementary Fig. 8. Effects of miR-10b and U6 snRNA on CDC42 alternative splicing. a Differential stability of CDC42 iso-1 and iso-2 variants. LN229 cells have been treated with Actinomycin D followed by qRT-PCR analysis of CDC42-iso1 and CDC42-iso2 variants. b qRT-PCR analysis of CDC42-iso1 or CDC42-iso2 levels in GBM8 cells transfected with either U6 ASOs, miR-10b inhibitor, mimic, or corresponding control oligonucleotides (mean ± SD, n = 3). Fold-change in the expression of the isoforms is plotted relative to the corresponding control conditions. P values were calculated using two-tail unpaired t-test. c Western blotting analysis of the indicated proteins in GBM8 cells transfected with either U6 ASOs, miR-10b inhibitor, or mimic. * P < 0.05; ** P < 0.01. [file 12943_2022_1494_MOESM8_ESM.tif]

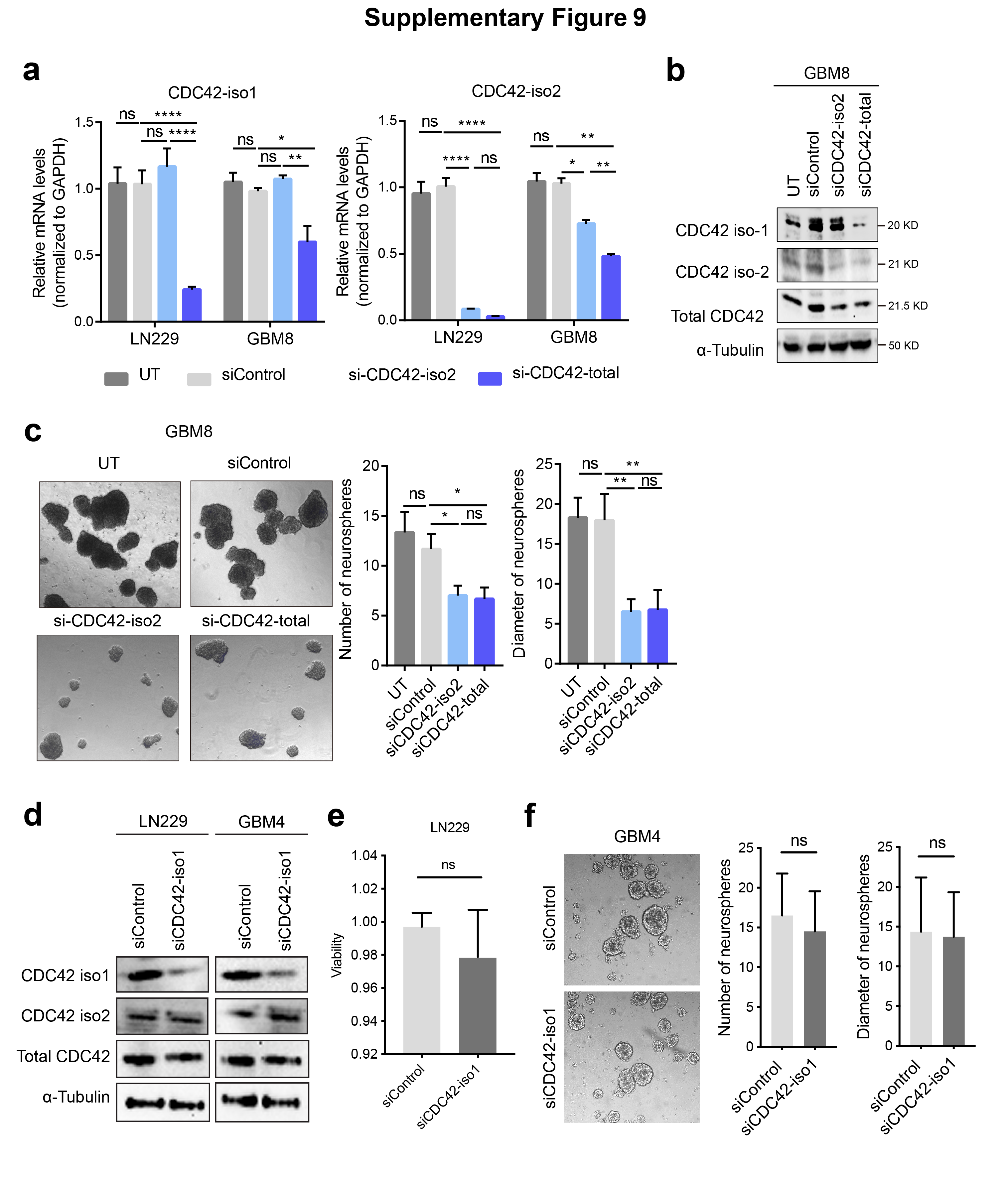

Supplement: Supplementary file 9 — Additional file 9 Supplementary Fig. 9. CDC42 promotes the growth of GSCs. a GBM8 cells were transfected with siCDC42-iso2, siCDC42-total, or control siRNAs, followed by the qRT-PCR analysis. b GBM8 cells were transfected with siCDC42-iso2, siCDC42-total, or control siRNAs, followed by Western blotting analysis. KD of the CDC42-iso2 reduced total CDC42 levels. c The growth of GSC spheroids, untreated or transfected with CDC42-targeting siRNAs, have been analyzed. The number and size of GSC neurospheres have been calculated at day 7 after transfections (mean ± SD, n = 3). P values were calculated using two-tail unpaired t-test. d LN229 and GBM4 cells were transfected with siCDC42-iso1 or control siRNAs, followed by Western blotting analysis. KD of the minor isoform CDC42-iso1 was insufficient to reduce total CDC42 levels. e Cell viability was monitored 72 h after transfection by WST-1 assay (mean ± SD, n = 3). P values were calculated using two-tail unpaired t-test. f The growth of GSC spheroids, transfected with siCDC42-iso1 or control siRNAs, have been analyzed. The number and size of GSC neurospheres have been calculated at day 7 after transfections (mean ± SD, n = 3). P values were calculated using two-tail unpaired t-test. * P < 0.05; ** P < 0.01; *** P < 0.001; ns, no significance. [file 12943_2022_1494_MOESM9_ESM.tif]

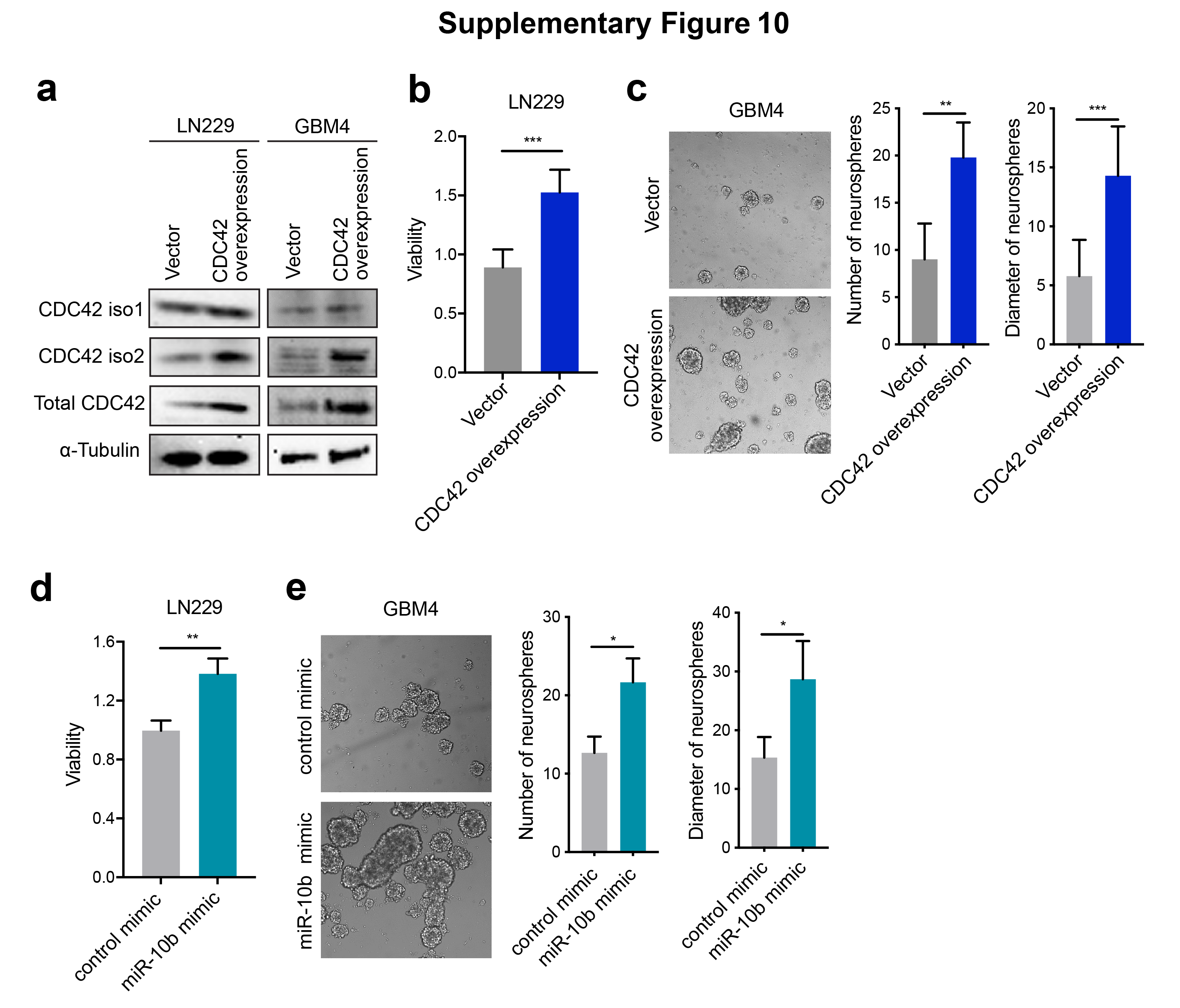

Supplement: Supplementary file 10 — Additional file 10 Supplementary Fig. 10. Overexpression of CDC42 iso-2 promotes glioma growth similarly to miR-10b. a LN229 and GBM4 cells were transfected with CDC42- overexpressing or control plasmid, followed by Western blotting analysis. b Cell viability was monitored 72 h after transfection by WST-1 assay (mean ± SD, n = 3). P values were calculated using two-tail unpaired t-test. c The growth of GSC spheroids has been analyzed; the number and size of GSC neurospheres have been calculated at day 7 after transfections (mean ± SD, n = 3). P values were calculated using two-tail unpaired t-test. d LN229 cells were transfected with miR-10b mimics, or corresponding control oligonucletides, followed by WST-1 assay (mean ± SD, n = 3). P values were calculated using two-tail unpaired t-test. e The growth of GSC spheroids, transfected with miR-10b mimics or corresponding control oligonucletides, has been analyzed. The number and size of GSC neurospheres have been calculated at day 7 after transfections (mean ± SD, n = 3). P values were calculated using two-tail unpaired t-test. * P < 0.05; ** P < 0.01; *** P < 0.001. [file 12943_2022_1494_MOESM10_ESM.tif]
